# A layered analysis of self-explanation and structured reflection to support clinical reasoning in medical students

**DOI:** 10.1007/s40037-020-00603-2

**Published:** 2020-07-30

**Authors:** Martine Chamberland, Silvia Mamede, Linda Bergeron, Lara Varpio

**Affiliations:** 1 Department of Medicine and Centre de Pédagogie des Sciences de la Santé, Université de Sherbrooke, Sherbrooke, Québec Canada; 2 Institute of Medical Education Research Rotterdam, Erasmus Medical Center, Erasmus University, Rotterdam, The Netherlands; 3 Centre de Pédagogie des Sciences de la Santé, Université de Sherbrooke, Sherbrooke, Québec Canada; 4 Department of Medicine, F. Edward Hébert School of Medicine and Uniformed Services University of the Health Sciences, Bethesda, MD USA

**Keywords:** Layered analysis, Self-explanation, Structured reflection, Clinical reasoning

## Abstract

Self-explanation and structured reflection have been studied independently with results suggesting that both learning interventions can effectively support medical students’ clinical reasoning development. Given this evidence, medical schools may want/begin to implement these interventions in their curricula. Implementing educational interventions requires educators to maintain the core philosophy and principles of the interventions intact while adjusting implementation techniques to the specificities of individual learning contexts. Educational scholars have yet to explicitly articulate the philosophy, principles and techniques of self-explanation and structured reflection. Without such descriptions, educators risk failing to realize self-explanation’s and structured reflection’s effect to support students’ clinical reasoning skill development in their implementations. Relying on the layered analysis approach, we articulate the philosophy, principles and techniques of self-explanation and structured reflection. This description is framed within the context of an actual implementation to illustrate the philosophies underpinning self-explanation and structured reflection, the principles that realize those philosophies, and the techniques that can be used to enact those principles. Building on the similarities between self-explanation and structured reflection, while also harnessing their differences, we identify why and how these interventions can be combined in a single implementation, while preserving their philosophies and principles. The layered analysis of self-explanation and structured reflection offers essential insights into the underpinnings of these interventions. They are articulated in this manuscript in hopes that other scholars will continue to refine these descriptions thereby facilitating effective use of self-explanation and structured reflection for clinical reasoning development.

**Electronic supplementary material:**

The online version of this article (10.1007/s40037-020-00603-2) contains supplementary material, which is available to authorized users.

## Introduction

Helping students to build deeply understood, interconnected knowledge is a central objective for fostering clinical reasoning skills [1]. Self-explanation and structured reflection are two interventions aimed at such knowledge building among students [2]. Self-explanation involves having a learner work individually and independently through learning materials by explicitly generating explanations to him/herself that deepen understanding [3]. Structured reflection consists of requesting students to compare and contrast plausible diagnoses for clinical cases with the aim of fostering the refinement of illness scripts stored in students’ memories. In this article, we describe in detail self-explanation and structured reflection as learning activities that can be implemented in a medical curriculum. We do this through layered analysis [4], which we briefly describe, and which provides a way of representing conceptually an educational intervention. Finally, we present how self-explanation and structured reflection might be combined and illustrate this combination through an example of how we implemented both learning activities in an undergraduate medical program.

## Research on self-explanation and structured reflection in medical education

Self-explanation and structured reflection have been studied independently as interventions to improve medical students’ clinical reasoning. In experimental research where students engaged in a learning activity followed one week later by an assessment phase, learners who used self-explanation in the learning phase had a better diagnostic performance on assessment than learners who did not use self-explanation. This effect was observed on less familiar topics [5] for which students expressed more biomedical knowledge while self-explaining [6]. In another experimental study, students’ diagnostic performance was further improved when, in addition to self-explanation, they were exposed to a near-peer self-explanation example and to prompts to process the example in the learning phase [7, 8]. Research on structured reflection has also adopted an experimental paradigm, typically including a learning session in which students diagnose clinical cases of similar-looking diseases either by a structured reflection procedure or conventional approaches such as making a differential diagnosis, and a delayed test session that requires all students to diagnose new cases of the same (or related) diseases. Students who practiced with structured reflection outperformed the other groups when diagnosing cases of the same [9, 10] and of related [10] diseases in the test. Furthermore, contrary to expectations, structured reflection has proved as effective as other approaches that provided students with additional knowledge such as studying examples of experts’ reasoning [11, 12].

Summarizing the literature on teaching interventions to support clinical reasoning in medical students, Schmidt & Mamede [2] concluded that, based on experimental evidence, self-explanation and structured reflection are valuable strategies for supporting a knowledge-oriented approach to learning clinical reasoning. With this experimental evidence suggesting that these interventions can help improve students’ clinical reasoning skills, implementing self-explanation or structured reflection in authentic educational practices is a logical next step. However, as other researchers have pointed out, implementing educational interventions is a complex challenge [13]. Indeed, this requires educators to keep the core philosophy and principles of interventions intact while adjusting implementation techniques to the specificities and affordances of individual learning context [4, 14].

The alignment between an intervention’s philosophy, principles and techniques can be explored and confirmed by asking manipulation check questions such as: What are the underpinning principles and philosophy of the intervention? Was our intervention truly an implementation of that innovation [4, 13, 14]? To answer such questions, we have to be able to move our focus beyond the techniques of *how* the implementation was achieved, to include understanding the underpinning philosophy and upholding principles that support *why* those techniques are used.

## The layered analysis: A summary

Cianciolo and Regehr’s layered analysis provides a way of “representing educational intervention in a conceptually meaningful way.” [4, p. 790]. The layered analysis approach unpacks the intervention into three different layers:

1. *The philosophy* at the core of the intervention, defined as “the foundational layer, the essence of an intervention. It is a context-independent, idealized statement of the learning conditions that must hold for the intervention to be what its designer claims it is” [4, p. 790] ;
2. *The principles *that bring the philosophy to life, defined as “the structural aspects of the intervention, which may be adjusted to context, but nevertheless represent somewhat generalized and relatively stable approaches to establishing conceptual learning conditions” [4, p. 790];
3. *The techniques *that realize the principles, defined as aspects of the intervention that are “the most context-sensitive, which account for contextual factors and allow the intervention to be tailored to a local setting.” [4, p. 790].

This layered analysis identifies critical elements of the intervention (i.e., its philosophy and principles) that must be upheld through the implementation, as well as the elements (i.e., the techniques) that may be modified to adapt to local contextual conditions without jeopardizing the intended action.

This paper presents a layered analysis of self-explanation and structured reflection in hopes that this description will facilitate the use of educational interventions in support of the development of diagnostic reasoning skills in medical students. We begin this analysis by presenting the philosophy and principles of each intervention, discussing how they overlap and how they are different. The large-scale implementation of self-explanation and structured reflection at a medical school is used to provide an illustrative example of the *technique* layer; that is, one possible way of operationalizing self-explanation and structured reflection to support clinical reasoning development in undergraduate medical education.

## Self-explanation: A layered analysis

Definition: self-explanation requests students to explain to themselves the underlying mechanisms of signs and symptoms in a to-be-solved clinical case in the purpose of deepening their understanding. This strategy helps students make links between different pieces of information through knowledge elaboration and knowledge integration, while simultaneously monitoring their evolving understanding. Further, self-explanation requires students to revise their knowledge structures [3]. Self-explanation has been studied in a variety of domains [3, 15–20], and in medicine to support clinical reasoning [21]. Using self-explanation while solving clinical cases for developing clinical reasoning helps students link biomedical knowledge and/or underlying principles to clinical features [5, 6].

### Underpinning philosophy of self-explanation in medicine

There are two theories underlying self-explanation in medicine: generative learning theory and the theory of expertise in medicine. Generative learning theory aligns within the cognitive views of learning, in particular contemporary constructivist theories. Generative learning theory proposes that “learning involves actively constructing meaning from new, to-be-learned information by mentally reorganizing it and integrating it with one’s existing knowledge.” [22]. According to this theory, meaningful learning (i.e., developing a deep understanding of the material that can be further applied to new contexts) relies on students’ engagement with and cognitive processing of information during learning. Appropriate cognitive processing involves selecting, organizing (i.e., building internal connections) and integrating the new information with prior knowledge (i.e., building external connections). Principle-based self-explanations, which are elaborations generated by the student that explain an element of the problem in reference to an underlying principle of the domain, appear the critical and most powerful ones [15]. By drawing their own interpretations and by generating inferences from the material—principle-based self-explanations, in particular—students process, construct, and revise their mental models [15, 22]. In these ways, self-explanation seeks to promote generative learning [3, 22, 23].

The theory of expertise acquisition in medicine contends that the development of clinical reasoning evolves through four stages of knowledge development and organization [1, 24]. In the first stage, the student constructs elaborate causal networks in which biomedical knowledge is represented. Next, in the second stage, knowledge becomes progressively compiled into a limited number of concepts which incorporate prior networks (i.e., knowledge encapsulation). In the third stage, illness scripts emerge. An illness script is a mental representation of a disease that comprises mainly clinical knowledge represented by the enabling conditions (i.e., factors that facilitate the occurrence of the disease) and the consequences of the disease in terms of complaints, signs and symptoms [18]. Encapsulated biomedical knowledge provides the faults (i.e., a brief description of the malfunction) and increases the coherence of the script [1, 25]. With clinical exposure, this formal knowledge is enriched, in the fourth stage, by specific patient examples (i.e., instances). In the process of diagnostic reasoning, clinicians activate relevant illness scripts, comparing and contrasting alternative scripts to find the best match with the specific case.

Growing out of these foundational theories, the philosophical underpinning of self-explanation proposes that important learning happens when an individual student activates his/her current knowledge base by systematically engaging with learning materials that somewhat exceed the learner’s current skill level (i.e., materials that challenge the learner but do not so far surpass the learner’s knowledge base so as to be unachievable). In these situations, the learner is prompted to build on his/her knowledge base (i.e., biomedical and clinical knowledge), to deepen his/her understanding, and to identify personal knowledge gaps.

### Principles arising from this philosophy

We propose that seven principles arise from this philosophy, enabling it to be realized in an educational intervention. These principles recognize that self-explanation:

1. Is an individual, learner-centered learning activity;
2. Requires prior knowledge activation;
3. Requires the learner to elaborate on current knowledge while working with the learning material;
4. Requires the learner to link elements of the learning material or prior knowledge to underlying principles (i.e., biomedical knowledge) to deepen his/her understanding;
5. Requires the learner to analyze the problem in a systematic and deliberate way;
6. Allows the learner to monitor the state of his/her knowledge, becoming aware of gaps and ambiguities in his/her knowledge;
7. Requires the learning material to be sufficiently challenging to require the individual to engage in deep knowledge building.

## Structured reflection: A layered analysis

Definition: Structured reflection requires students to generate alternative diagnostic hypotheses for a clinical case and systematically identify, from the case, findings in favor, against, and missing for each diagnosis under consideration [9].

When students use structured reflection for developing clinical reasoning, they engage in elaboration and refinement of the illness scripts that they have stored in memory. Because these mental representations of diseases are critical for future problem solving, [1] structured reflection has the potential to improve students’ diagnostic competence.

### Underpinning philosophy of structured reflection

Structured reflection is informed by the model of reflective practice in medicine and, similar to self-explanation, the theory of expertise development in medicine. Research on reflective practice in medicine has led to the development of a 5-factor model: deliberate induction, deliberate deduction, testing and synthesizing, openness for reflection and meta-reasoning [26]. Structured reflection was initially conceived and tested as an approach to improve physicians’ diagnostic performance and reduce diagnostic errors [27–29]. Subsequently, it has been investigated as an educational intervention. This latter research builds upon the assumption that allowing students to practice elements of the structure of reflective practice while diagnosing clinical problems—thereby elaborating upon and reconstructing their knowledge relevant to arrive at diagnosis—could promote the development of illness scripts. This claim, consistent with psychological research showing the benefits of comparing and contrasting cases for learning [30], has been supported by experiments with medical students [9, 10]. The theory of expertise in medicine has been discussed in the previous section on self-explanation [1, 24].

The philosophical core of structured reflection, emerging from these theoretical foundations, proposes that important learning happens when a student engages deliberately and systematically with a to-be-solved problem that is appropriate to the student’s level, by comparing and contrasting alternative diagnoses, thereby allowing the student to build (and/or refine or consolidate) illness scripts. Structured reflection is aligned within the constructivist tradition assuming that people actively try to organize and make sense of the information that they encounter, thereby building knowledge in idiosyncratic ways, and focusing attention on mental representations of knowledge [31].

### Principles arising from this philosophy

We propose five principles that arise from this philosophy, enabling it to be realized through an educational intervention. These principles recognize that structured reflection:

1. Is a learner-centered learning activity;
2. Requires the learner to work with a clinical case to be solved;
3. Engages the learner in using induction followed by deduction and testing while deliberately reflecting on plausible diagnoses, comparing and contrasting their respective illness scripts;
4. Requires the learner to go systematically through a number of actions while working through the clinical case:
  - Generate an initial diagnosis for the case;
  - Identify the findings in the case that: support the diagnosis; speak against the diagnosis; should be present if the diagnosis was correct but are absent in the case;
  - Based on the confrontation with contradictory evidence emerging from the previous analysis, list plausible alternative diagnoses;
  - Perform the same analysis for each diagnosis;
  - Prioritize the diagnoses in terms of likelihood;
5. Requires that the clinical case be appropriately targeted to the learner’s level of expertise (i.e., the case should address content for which the learner has sufficient prior knowledge to reflect upon and is still at the stage of building/refining his illness scripts. In other words, the case represents a reasonable challenge).

## Self-explanation and structured reflection to support clinical reasoning development: similarities and differences

The philosophies and principles of self-explanation and structured reflection have several commonalities. Both are learner-centered and aim to support the student’s knowledge building. Both require students to engage systematically and deliberately with a clinical case, using and elaborating on prior knowledge while working through the possible diagnostic solutions. However, the specific type of knowledge that the student works with is different [2]. Self-explanation prompts the student to generate explanations for the clinical data presented in the case, thereby encouraging the learner to make links between clinical knowledge and biomedical knowledge. In contrast, structured reflection requires the student to compare and contrast the clinical features of alternative diagnoses, thereby targeting the development of clinical knowledge. As long as the student’s prior knowledge is appropriate to the task, self-explanation and structured reflection support the development of illness scripts in complementary ways: self-explanation fosters coherence of the script with encapsulation of biomedical knowledge and enrichment of the faults component of the script, and structured reflection supports refinement of the clinical components (e.g., enabling conditions, symptoms and physical signs). Given this, we contend that self-explanation and structured reflection are complementary and can be implemented together in an educational intervention.

## Self-explanation and structured reflection techniques: an illustration from a large-scale implementation

To illustrate the techniques that we employed to implement self-explanation and structured reflection, we provide an example of a specific educational intervention that was implemented in an undergraduate medical program. The similarities and differences between self-explanation and structured reflection, and thus their complementarity described earlier, provided the rationale for combining and sequencing self-explanation and structured reflection within the same learning activity. This combined SE-SR activity was designed for and as a part of a broader renewal of a 4-year undergraduate competency-based medical program at the Université de Sherbrooke, Québec, Canada.

The curriculum is designed around a series of professional clinical situations of increasing complexity, divided into five blocks of activities per year for the first 2 years of undergraduate medical education. Each block lasts between 6–8 weeks and comprises a variety of activities through which students progressively acquire specific knowledge from basic sciences to problem management for a number of clinical situations. At the end of each block, an integration week offers opportunities for students to deepen and apply their newly acquired knowledge. The SE-SR activity is part of this integration week. The activity consists of a web-based, 90-minute learning session that students complete individually. Before the first session, students have to review three training materials: conceptual information on clinical reasoning; descriptions of self-explanation and structured reflection (i.e., why and how this activity will contribute the development of their clinical reasoning skills); and an audio recorded example of a student engaged in self-explanation and structured reflection with a specific problem and a clinical case. During each session, three challenging clinical cases relevant to the particular block of activities are presented to the student. Appendices 1, 2 and 3 of the Electronic Supplementary Material present an example of a clinical case, illustrations of a student’s self-explanation, and a student’s structured reflection completed grid for the case, respectively. With a specific time constraint (30 min per case), students solve each case using both these strategies. First, they verbally review the case using self-explanation to the point of providing a diagnosis; this verbalization is audio-recorded. Prompts for self-explanation are incorporated within each case. They then use structured reflection by completing a grid comparing and contrasting three plausible diagnostic hypotheses they were considering. After each case, students immediately receive feedback in the form of a completed structured reflection grid. The SE-SR activity recurs ten times over the first two years.

**Fig. 1:**
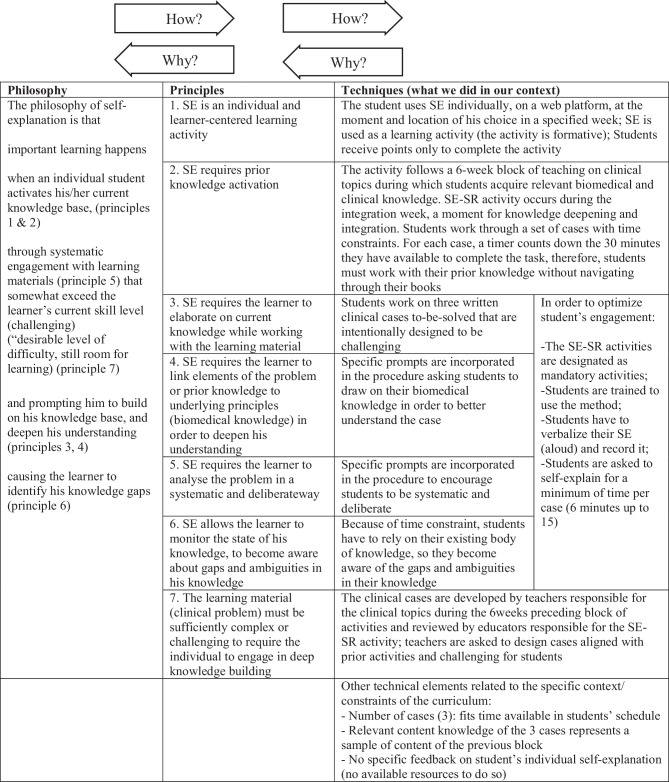
Philosophy, principles and techniques of self-explanation (SE)

**Fig. 2:**
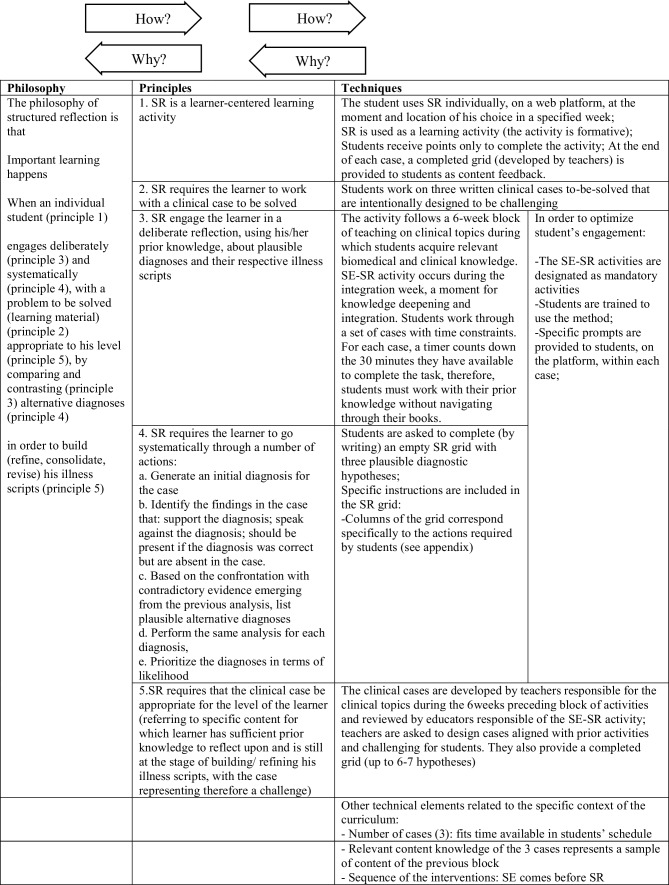
Philosophy, principles and techniques of structured reflection (SR)

Figs. 1 and 2 offer detailed illustrations of the techniques used in this implementation. These tables also illustrate how all the instructional techniques align with and support the philosophy and principles of self-explanation and structured reflection.

## Discussion

Using a layered analysis [4, 14], we were able to identify the philosophy, principles, and techniques of self-explanation and structured reflection, and to combine them into a single intervention.

We found that the layered analysis framework greatly supported our ability to translate educational theory into practice. For instance, the self-explanation literature is quite abstract and is scattered across different disciplines [15]; however, the effectiveness of self-explanation has been robustly determined across these disciplines [15, 22, 32]. The challenge for medical educators has been to implement self-explanation in ways that aligned teaching techniques to self-explanation’s philosophy and principles. Layered analysis supported our planning of the self-explanation activity, our revisions to those designs after it was implemented, and our implementation evaluation plan. Unpacking an intervention into its different layers allows us to examine our education innovation’s “intended function” [4, p. 790] in our specific context.

We suggest that a means of testing the alignment of an intervention’s implementation techniques with its philosophy and principles is to use *how* and *why* questions [33] (see Figs. 1 and 2 for an illustration). To ensure that our techniques were aligned with the intervention’s principles and philosophy, we would ask: “*Why* should this technique be used?” If we could answer that question by tying a technique back to a principle and philosophy, then we were confident of the technique’s alignment. If we could not create that connection, then we concluded that the technique either needed to be modified to realize the principles and philosophy of self-explanation and/or structured reflection, or it needed to be abandoned. To ensure that self-explanation and/or structured reflection’s philosophy and principles were enacted in our techniques, we would ask: “*How *is this principle/philosophy realized in this innovation?” If we could answer this question by tracking an element of the philosophy through to a principle and then to a technique we were assured that the intervention aligned with the foundations of self-explanation and/or structured reflection. If we were unable to complete that tracking, then techniques needed to be developed or modified to answer the *how *question.

Deliberately using layered analysis to describe our educational intervention also helped us to justify the combining and sequencing of our self-explanation and structured reflection interventions. This analysis highlighted where self-explanation and structured reflection dovetailed together well, and how they could be sequenced to best complement each other. Requiring students to first use self-explanation on a clinical case allowed them to analyze, to elaborate, and to try to understand more deeply the problem by linking clinical to biomedical knowledge, as well as to generate a variety of diagnostic explanations. This work prepared the learners for the subsequent structured reflection activity on that case.

Furthermore, the layered analysis usefully enabled us to monitor our implementation and refine it to ensure that it realized the foundational philosophy and principles of self-explanation and/or structured reflection. Data from the monitoring of the implementation are of particular importance because, when implemented in authentic settings, new constraints or opportunities in the local context often emerge requiring modification of the intervention. Layered analysis can help identify when those constraints and opportunities are impacting the alignment of the interventions’ philosophy, principles, and techniques. This is essential information that supports the implementation team’s ability to develop and justify appropriate technical changes that reinforce (or at least do not undermine) the underlying principles. For example, in response to monitoring data, we have expanded the duration of the period in which students could complete the SE-SR session at the end of the block of activities from 3 to 10 days. This change seems to have reinforced students’ engagement by allowing them to choose the moment to complete the activity (Principle one of self-explanation and structured reflection: learner-centered activities). We have also modified 2 cases out of 30 which were judged exceedingly difficult (Principle 7 of self-explanation and principle 5 of structured reflection).

Layered analysis of self-explanation and structured reflection and appropriate monitoring of its implementation also enriched our understanding of ways our techniques were harnessed by the learners. Formal analysis of the data collected for monitoring the implementation is not completed yet. Nevertheless, the layered analysis allowed us to notice signs of ways learners were using the activity. For example, we noted that some students engaged in the activity early in the dedicated period of time, taking advantage of the effect on monitoring of knowledge to orient their subsequent studies. In contrast, other students preferred to use the SE-SR strategy near the end of the period, allowing them time for revision of specific content before engaging with the case. The flexibility of the schedule allowed students to use the activity in ways that best suited their individual needs (Principle 1 of self-explanation and structured reflection: learner-centered activities).

Our descriptions of self-explanation and structured reflection used to support students’ clinical reasoning is a proposition: we acknowledge this is one way to describe them, not the only way. However, our team includes scholars who have developed and worked with self-explanation and structured reflection extensively. Therefore, we are confident of our descriptions but welcome the opportunity to see how others conceive of and implement self-explanation and structured reflection. This is perhaps the greatest opportunity offered by layered analysis: it provides scholars with premises and propositions that can be tested and examined by others so that we can collaboratively engage in the development and refinement—and perhaps even the abandonment—of different educational interventions. Historically we have described our techniques in publications. Now we have the opportunity to look beyond the techniques and into the philosophies and principles that stand behind them. These are the elements of an educational intervention that can be transferred across contexts, even when the techniques cannot be.

## Caption Electronic Supplementary Material

Appendices
