# Supplementary material for: A layered analysis of self-explanation and structured reflection to support clinical reasoning in medical students
Source: Perspect Med Educ. 2020 Jul 30;10(3):171–9. doi: 10.1007/s40037-020-00603-2 (PMC8187699; doi:10.1007/s40037-020-00603-2)
Supplement: Supplementary file 1 — Appendices [file 40037_2020_603_MOESM1_ESM.docx]

Appendix 1

Example of a clinical case used for SE-SR

A 68-year-old woman presents with jaundice.

**HPI:** The patient noted a gradual yellow-green discoloration of her skin over three weeks. She noticed that her urine have become dark, and her stools have become pale but she reports no diarrhea or constipation. She has no abdominal pain other than a vague epigastric discomfort which she has experienced after meals. Her appetite seems to be diminished and she reports having lost 5-7 kg over the past month. She denies fever, chills, or night sweats. There is no travel history and no change in his dietary habits.

**PMH:** Hypertension. One episode of acute diverticulitis in 2006. Superficial thrombophlebitis of the left lower extremity 2 months ago.

**Medications:** Hydrochlorothiazide 12, 5 mg QD

**Habits:** A retired secretary, she still lives in her own house. There is no history of smoking or of alcohol use.

**Physical examination:** The patient’s general appearance is normal except for the presence of a deeply jaundiced skin, BP 150/85, Pulse 78, afebrile, weight 60kg. There is no enlargement noted in any of the lymph node groups. Cardiac examination is within normal limits. Lung examination is normal. On abdominal examination, there is no tenderness noted. The liver and spleen are not enlarged. There are no palpable masses. The rectal examination is normal. There is no edema of the lower extremities.

**Laboratory investigations :** CBC : hemoglobin 115 g/L(120-160) MCV 88 fL(80-100) WBC 6,2 X 10^9^/L(3,8-10,6) Platelets 350 X10^9^/L(130-400); Serum electrolytes and creatinine are normal; Fasting blood sugar is 6,9 mmol/L(3,3-6,1); AST 52 IU/L (14-50) , ALT 75 IU/L (21-72), alkaline phosphatase 625 IU/L(43-200), total bilirubin 110umol/L (3,4-17); direct bilirubin 100umol/L; albumin 33g/L (35-50); INR and PTT normal. Urinalysis: the urine is brown, testing strongly positive for bilirubin, and negative for urobilinogen.

Appendix 2

Illustration of a student’s self-explanation statements

A 68-year-old woman presents with jaundice.

**HPI:** The patient noted a gradual yellow-green discoloration of her skin over three weeks. She noticed that her urine have become dark. *It might be because there is more bilirubin in the urine.* She has no abdominal pain other than occasional vague epigastric discomfort which she has experienced after meals. *Well, that doesn’t sound very specific…it might be related to a problem with the liver? Impaired metabolism or secretion of the bilirubin.* Her stools have become pale but she reports no diarrhea or constipation. *Sometimes in jaundice, the stools become more pale… in fact when there is no bilirubin …when there is like an obstruction in the biliary track. So it might be the case here… but I don’t understand why she has no pain…Not a gallstone?* Her appetite seems to be diminished and she reports having lost 5-7 kg over the past month. *Significant loss of weight…So, she has probably ate less in the past three weeks. I don’t think that food malabsorption could explain the weight loss, because she has no diarrhea. Also… weight loss may be a symptom of cancer.…*She denies fever, chills, or night sweats. *Since these symptoms are absent, I presume that inflammation or infection such as a viral hepatitis is a less probable process involved here… however I am not sure if there is always fever with viral hepatitis. I have to check that.* There is no travel history and no change in his dietary habits.

Appendix 3

Illustration of a structured reflection grid completed by a student

| **Order of Probability** | **Diagnosis** | **Elements in favor** | **Elements against** | **Elements expected but missing** |
| --- | --- | --- | --- | --- |
| 1 | Pancreas cancer | Jaundice;  Subacute;  No pain;  Anorexia;  Weight loss;  Dark urine;  Pale stools;  Direct bilirubin increased |  | Dull epigastric pain;  Recent onset diabetes |
| 2 | Choledocholithiasis | Jaundice;  Dark urine;  Pale stools;  Direct bilirubin increased | Subacute;  No pain;  Significant weight loss;  No fever | Biliary colic;  Nausea, vomiting |
| 3 | Viral Hepatitis | Jaundice;  Subacute;  Dark urine;  Anorexia | Pale stools;  No fever, chills, night sweat | Risk factor (travel, iv drugs, sexual contact);  Right upper quadrant tenderness;  AST-ALT increased |
